# *Staphylococcus lugdunensis* Bacteremia with an Infected Aortic Thrombus in a Preterm Infant

**DOI:** 10.3390/children9010046

**Published:** 2022-01-02

**Authors:** Srinivasan Mani, Praveen Chandrasekharan

**Affiliations:** 1 Division of Neonatology, Department of Pediatrics, ProMedica Russell J. Ebeid Children’s Hospital, Toledo, OH 43606, USA; 2 Division of Neonatology, Department of Pediatrics, University at Buffalo, Buffalo, NY 14260, USA; pkchandr@buffalo.edu

**Keywords:** *Staphylococcus lugdunensis*, infant, thrombus

## Abstract

*Staphylococcus lugdunensis* is a rare cause of late-onset sepsis in preterm infants. To our best knowledge, we report the fourth case of a male preterm infant who developed fulminant late-onset sepsis due to *Staphylococcus lugdunensis* with persistent bacteremia secondary to an infected aortic thrombus confirmed with two positive blood cultures. Our patient was an extremely low birth weight growth-restricted infant born at 27 weeks gestation and initially required an umbilical arterial catheter for blood pressure and blood gas monitoring. The course of this neonate was complicated by severe respiratory distress syndrome that evolved into chronic lung disease along with multiple episodes of tracheitis. Hemodynamically, the infant had a significant patent ductus arteriosus, and an episode of medical necrotizing enterocolitis followed by *Staphylococcus lugdunensis* septicemia. He was diagnosed with an infected aortic thrombus, probably the occult focus responsible for the persistent bacteremia. After a 6-week course of intravenous antibiotics and 4-week course of anticoagulant therapy, the infant responded and recovered without complications.

## 1. Introduction

Coagulase-negative Staphylococcus is the most common cause of late-onset sepsis in newborn infants in intensive care units [1]. *Staphylococcus lugdunensis* is one of the 40 known coagulase-negative staphylococci (CoNS) [2]. *S. lugdunensis* and *S.schleiferi* were discovered as a novel species at the French National Reference Center for Staphylococci, Lyon, France, by deoxyribonucleic acid relatedness studies in 1988 [3]. They were classified as CoNS with a unique ability to cause a deadly form of native valve endocarditis similar to Staphylococcus aureus. The first case of native valve endocarditis due to this opportunistic pathogen in the pediatric population was reported in 2002 [4]. *S. lugdunensis* is a typical skin commensal that preferentially colonizes the body parts with apocrine sweat glands, like the perineal region, axilla, auditory canal, eyelids, and mammary areola [5]. Colonization of intestines is also known. The colonized body parts’ skin and soft tissue infections are the most common infections due to *S. lugdunensis*. The predilection for each site varies with age. *S. lugdunensis* have been reported to cause many other infections like native and prosthetic valve endocarditis, osteomyelitis, prosthetic joint infections, endophthalmitis [6], foreign body-associated infection involving ventriculoperitoneal shunts [7], peritoneal dialysis catheters [8], hemodialysis catheters, and external ventricular drains [9].

We report a preterm infant who developed fulminant late-onset sepsis due to *S. lugdunensis* with persistent bacteremia due to an infected aortic thrombus. The infant described in this report was born in our facility and managed in a level IV neonatal intensive care unit at a regional perinatal center with more than 850 admissions and more than 3000 live births annually.

## 2. Case

An intrauterine growth-restricted premature baby boy was born to a 25-year-old mother. A notable finding during the pregnancy was elevated umbilical artery Doppler flow. Previous pregnancy was complicated by preeclampsia. The mother received prenatal vitamins and aspirin. The infant was delivered by emergent cesarean section for non-reassuring fetal heart rate under general anesthesia after completing a course of antenatal steroids. The rupture of membranes was at delivery with clear amniotic fluid. The infant was born at a gestational age of 27 weeks + 2 days with a weight of 0.723 kg (10.4 percentile), length of 31.0 cm (2.9 percentile), and head circumference of 23.5 cm (14.9 percentile). At birth, he was apneic, bradycardic, and floppy, requiring intubation and positive pressure ventilation. The APGAR scores were two at 1 min and seven at 5 min. Secondary to bradycardia, the umbilical cord was cut immediately at birth.

### 2.1. NICU Course

On admission to the NICU, the infant presented with respiratory distress syndrome and systemic hypotension requiring exogenous surfactant and a normal saline fluid bolus. The infant had an umbilical arterial catheter (UAC) for invasive blood pressure monitoring and blood gas sampling for 5 days after birth. Our patient received respiratory support with conventional mechanical ventilation. He required two additional doses of surfactant and remained intubated on mechanical ventilator support. On the fourth day of life (DOL), the infant’s respiratory status worsened, requiring transition to high-frequency jet ventilation. On DOL10, the infant was diagnosed with tracheitis due to methicillin-resistant *Staphylococcus epidermidis* (MRSE) and responded to the appropriate antibiotic course. On DOL22, an echocardiography obtained for a systolic murmur heard on auscultation along with worsening respiratory status revealed a hemodynamically significant patent ductus arteriosus (HS- PDA), which responded to an extended course of indomethacin therapy. On DOL28, he was diagnosed with another episode of tracheitis with *Klebsiella oxytoca* and recovered with an excellent response to appropriate antibiotics. On DOL31, the baby was transitioned to noninvasive respiratory support and later weaned to room air by DOL63 while on inhalational steroids and caffeine.

The enteral feed advancement was interrupted frequently due to bowel dysmotility. Presumed to have milk protein intolerance, he was transitioned to a hydrolyzed formula. After weaning parenteral nutrition off on DOL41, he tolerated full enteral feeds, and calories were increased to 27 kilocalories/ounce formula to improve growth. On DOL60, he had bloody stools, and an abdominal X-ray was suspicious for pneumatosis intestinalis. The feeds were stopped and he was placed on parenteral nutrition and bowel rest. The infant completed a 7-day course of vancomycin, gentamicin, and metronidazole and showed clinical improvement. We restarted the feeds on DOL68.

### 2.2. Staphylococcus lugdunensis Sepsis

On DOL71, he decompensated acutely with recurrent apneic episodes and hypotensive shock, requiring emergent intubation, mechanical ventilation, fluid resuscitation, and ionotropic support for a brief period. With the given clinical picture, bacterial sepsis was suspected. Laboratory investigation including blood and urine culture; a complete blood count with differential and C- reactive protein was sent. The feeds were stopped, and broad-spectrum IV antibiotics were started (vancomycin and ceftazidime). The laboratory parameters pointed towards bacterial sepsis (see Table 1).

**Figure 1:**
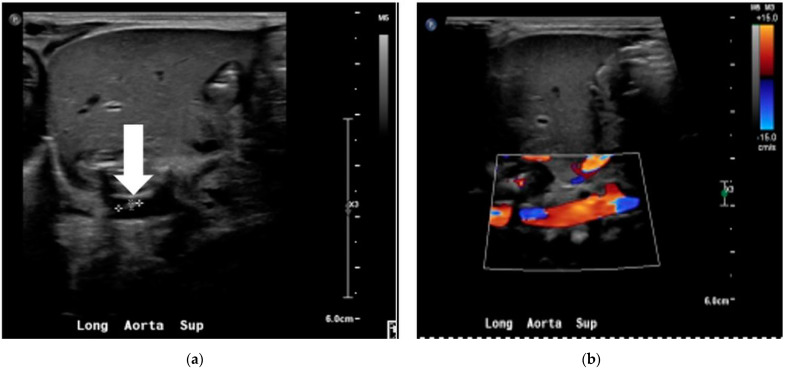
Aortic doppler study showing the non-occlusive thrombus in the grayscale (**a**) and color doppler (**b**).

We researched further to find an occult focus because of persistent bacteremia. Transthoracic echocardiogram, abdominal ultrasound, and head sonogram were normal. The aortic Doppler study revealed a non-occlusive thrombus (4 × 1 × 1.5 mm) in the infrarenal portion of the abdominal aorta (see Figure 1).

The antibiogram of the isolated bacterial strains was reviewed (see Table 2). Ceftazidime was discontinued, and vancomycin was continued at therapeutic levels. The infant showed clinical improvement on D3 of illness, and respiratory support was weaned off and was placed back on room air. The fourth blood culture returned sterile, and we confirmed it with three consecutive negative cultures.

We consulted subspecialists from pediatric infectious disease and hematology. The aortic thrombus, probably from UAC placement, was considered to be the infective focus responsible for the persistent bacteremia, and the infant was treated with a 6-week course of vancomycin. He was also started on enoxaparin, and the thrombus was followed with serial aortic doppler imaging.

**Figure 2:**
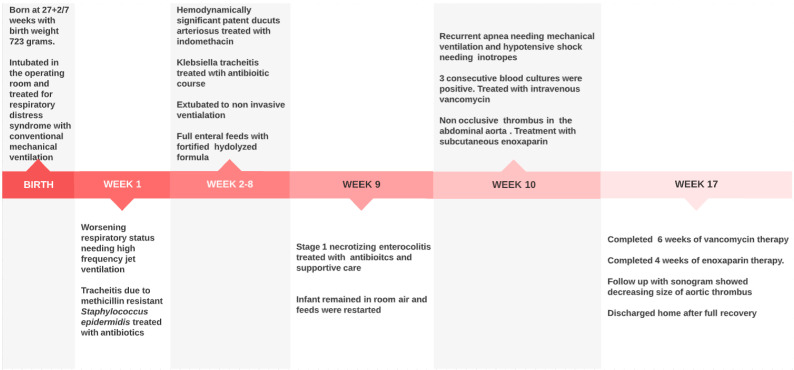
Sequential timeline of illness presentation.

The infant completed 6 weeks of vancomycin therapy and completed 4 weeks of enoxaparin therapy without complication. Follow-up aortic sonogram with dopplers showed a decrease in the size of the thrombus. As a result, the infant was discharged home with multispecialty outpatient follow-up. Figure 2 summarizes the timeline of the events described above.

## 3. Discussion

*S. lugdunensis* is a gram-positive, catalase-positive, coagulase-negative coccus, as well as a nonmotile, facultative anaerobe [10]. *S. lugdunensis* is rare in its occurrence, accounting for 3.6% of all CoNS-positive clinical samples in the pediatric population [11]. The specific incidence of *S. lugdunensis* among the neonatal population is unknown, although CONS is the most prevalent infection among the VLBW infants in the neonatal intensive care setting. Our patient is the fourth case of *S. lugdunensis* sepsis in the neonatal population reported so far [12,13,14] (see Table 3). The uniqueness of our patient’s disease is due to the fulminant presentation with septic shock needing inotropes, as well as persistent bacteremia with an infected aortic thrombus probably from a previously placed UAC.

Microbiological identification of *S. lugdunensis* is challenged by traditional laboratory techniques. Although *S. lugdunensis* cannot produce secreted coagulase like *S. aureus*, they can harbor a membrane-bound coagulase (clumping factor) that can cause *S. lugdunensis* to be erroneously classified as *S.aureus* if the lab uses slide coagulase or rapid latex agglutination tests instead of a more specific tube coagulase test [15]. To overcome these pitfalls and increase the speed and accuracy of species identification and antibiotic susceptibility testing, most modern laboratories use fully automated systems based on broth microdilution techniques [16,17]. The 16S rRNA sequencing is considered the gold standard in the species identification of CONS. However, analysis of bacterial proteome by matrix-assisted laser desorption ionization time-of-flight mass spectrometry (MALDI-TOF MS) has emerged as a promising technology with similar efficacy to genetic identification methods [18,19]. Our laboratory uses VITEK^®^2 (bioMérieux SA, Marcy l’Etoile, France) fully automated ID/AST system as a first-line method. In addition, our lab uses the MALDI- TOF MS method for additional confirmation in doubtful cases. VITEK^®^2 system was used to identify *S.lugdunensis* in all three samples in our patient.

*S. lugdunensis* can cause a broad spectrum of diseases. The disease caused by *S. lugdunensis* differs from other CoNS infections in clinical presentation and treatment options. The clinical presentation resembles *S.aureus* with a propensity to cause life-threatening invasive disease in preterm infants. *S. lugdunensis* can cause aggressive infective endocarditis, central line-associated bloodstream infections, and skin and soft tissue infections [20,21]. Indwelling foreign bodies like CSF shunts, peritoneal catheters, vascular grafts, intracardiac patches, and prosthetic cardiac valves are the major risk factors for the infection due to this bacterium [7,8]. *S. lugdunensis* has unique mechanisms to overcome shear stress and adhere to the sites of vascular injury and implantable devices. VWF binding protein and fibrinogen binding protein help in this process [22,23].

After starting the right antibiotic based on the sensitivity pattern in the appropriate dosage, the presence of persistent or recurrent bacteremia should raise suspicion for an occult focus. Apart from the routine clinical evaluation aimed at finding the focus, one should consider infected thrombus as a possibility in the patient population at risk, like preterm infants [24]. The diagnosis of infected thrombus is primarily clinical and by Doppler ultrasound. Histopathology, which could be confirmatory, can be obtained if surgical management is warranted [25]. Our patient had consecutive blood cultures positive for S. lugdunensis, two of them were after the antibiotic was changed based on sensitivity. Infection and central lines are important postnatal risk factors for neonatal thrombosis. Platelet-triggered inflammation secondary to infection plays a vital role in pathogenesis [26]. Our patient did not have a clinical suspicion of an arterial thrombus before the infection. We speculate that the thrombus identified as the endovascular nidus could be a complication of UAC placement immediately after birth.

Central lines are the most common portals for *S. lugdunensis* bacteremia [27]. However, our patient did not have a central line during the onset of the disease. Entry of the organism into the bloodstream can occur from the respiratory tract of mechanically ventilated infants or the gastrointestinal tract of infants with necrotizing enterocolitis. Preterm infants have their gastrointestinal tracts colonized with invasive strains of CONS, which could translocate to the bloodstream [28]. Our patient was in room air but had an episode of NEC 10 days before the onset of *S. lugdunensis* bacteremia, which shows that translocation from the intestines could have been the likely portal of entry.

*S.lugdunensis* is peculiar among the CONS in that the methicillin resistance is infrequent, whereas it is almost universal in the more common *S.epidermidis*. The *mecA* gene confers methicillin resistance to CONS and coagulase-positive *Staphylococcus* aureus by encoding a transpeptidase, penicillin-binding protein 2A. The first report of the *S.lugdunensis* harboring the mecA gene came in 2003 [12]. The bacterial strain that infected our patient probably carried this gene, which resulted in methicillin resistance needing treatment with vancomycin.

Our patient received three courses of intravenous antibiotics for localized sepsis before the bacteremia due to *S.lugdunensis* as described in this report. However, none of them were bloodstream infections. Two of those infections caused by *S.epidermidis* and *Klebsiella oxytoca* were restricted to the trachea while being on prolonged invasive mechanical ventilation with a hemodynamically significant patent ductus arteriosus. The aforementioned complications are known risk factors for recurrent late-onset infection in extreme prematurity [29]. The prenatal factors, namely fetal growth restriction and maternal hypertensive disorder seen in our patient, have been associated with increased risk of late-onset sepsis [30]. The third course of antibiotics was for necrotizing enterocolitis stage 1 without a positive blood culture.

Recurrent infections with multiple organisms raise the suspicion of primary immunodeficiency disorders (PID) even in the extremely preterm population. Family history of immunodeficiency, genetic syndromes, and failure to thrive are the critical clues to identifying PID in newborns and children [31]. However, our patient did not have any family history of immunodeficiency or features suggestive of abnormality involving the phagocytic system like hepatosplenomegaly, lymphadenopathy, skin and soft tissue infections, and delayed separation of the umbilical cord. The newborn screen did not show any T-cell receptor excision circle (TREC) assay abnormality. Although TREC assay is not comprehensive for identifying all the PID, several genetic syndromes and medical conditions associated with immunodeficiency apart from severe combined immunodeficiency syndrome can be identified [32]. PID like congenital neutropenia; chronic granulomatous disease; leukocyte adhesion defect; and Toll-like receptor signaling defects, which could cause recurrent infections due to *Staphylococcus* spp., were not evaluated due to a lack of definitive clinical suspicion.

## 4. Conclusions

*S. lugdunensis* is an infrequent cause of late-onset sepsis in preterm infants. It causes an aggressive disease similar to *S. aureus*, so it should not be considered a contaminant without further evaluation. *S. lugdunensis* infection can occur due to translocation from the colonized gastrointestinal tract following an episode of necrotizing enterocolitis. *S. lugdunensis* has unique mechanisms to overcome shear stress that can cause an infective thrombotic focus. Physicians should consider the presence of methicillin resistance among *S. lugdunensis* while choosing empirical antibiotic therapy in a sick preterm infant.

## Figures and Tables

**Table 1:** Laboratory results of sepsis evaluation at the time of presentation of the clinical illness.

Cell Count	D71	D72	D73
White cell count (×10^9^/L)	4	5.2	5.3
Hemoglobin (g/dL)	10.1	13.3	11.6
Hematocrit (%)	32.0	39.1	34.8
Neutrophil segmented/100 leukocytes (%)	40	40	26
Neutrophil—Band/100 leucocytes (%)	14	24	14
Lymphocyte (%)	37	21	54
Monocyte (%)	8	13	6
Eosinophil (%)	1	0	0
Metamyelocyte (%)	0	2	0
Platelets (×10^9^/L)	100–149	86	87
C-reactive protein	13.52	106.57	51.25
Blood Culture	*Staphylococcus* *lugdunensis*	*Staphylococcus* spp.	*Staphylococcus* *lugdunensis*
Urine Culture	No growth		
Cerebrospinal fluid			No growth

**Table 2:** Antibiogram of the bacteria isolated from the patient.

	*Staphylococcus lugdunensis*		Coagulase-Negative *Staphylococcus* Species		*Staphylococcus lugdunensis*	
Drug	Susceptibility	MIC	Susceptibility	MIC	Susceptibility	MIC
Ampicillin/Sulbactam	R		R		R	
Cephazolin	R		R		R	
Clindamycin	R	≤0.25	R	≥8	R	≤0.25
Erythromycin	R	≥8	R	≥8	R	≥8
Gentamicin	S	≤0.5	R	≥16	S	≤0.5
Levofloxacin	S	≤0.12	R	≥8	S	0.25
Linezolid	S	1	S	2	S	1
Oxacillin	R	≥4	R	≥4	R	≥4
Rifampin	S	≤0.5	S	≤0.5	S	≤0.5
Tetracycline	S	≤1	S	≤1	S	≤1
Trimethoprim/ Sulfamethoxazole	S	≤10	R	≥320	S	≤10
Vancomycin	S	≤0.5	S	1	S	≤0.5

Table 2 shows the susceptibility pattern and minimum inhibitory concentration (MIC) values in microgram/milliliter of the bacteria cultured in our patient. “R” denotes resistance, and “S” denotes susceptible.

**Table 3:** Previously reported neonatal presentation of *Staphylococcus lugdunensis* sepsis.

Year of Report	GA	B.WT.	AGE	Clinical Presentation	Comorbidity	Antibiotic	Central Line	Recovery
2003 [12]	29 w	980 g	23 d	Bacteremia—Apnea	RDS, PDA and pulmonary hemorrhage	Vancomycin	Yes	Yes
2015 [13]	41 + 4/7 w	3284 g	18d	UTI -Fever with rash	none	Cefazolin	No	Yes
2018 [14]	Unknown	NA	1 d	Infective endocarditis	PROM	Nafcillin	No	Yes
